# Supplementary figures and images for: Impaired upper respiratory tract barrier function during postnatal development predisposes to invasive pneumococcal disease
Source: PLoS Pathog. 2024 May 8;20(5):e1012111. doi: 10.1371/journal.ppat.1012111 (PMC11078396; doi:10.1371/journal.ppat.1012111)

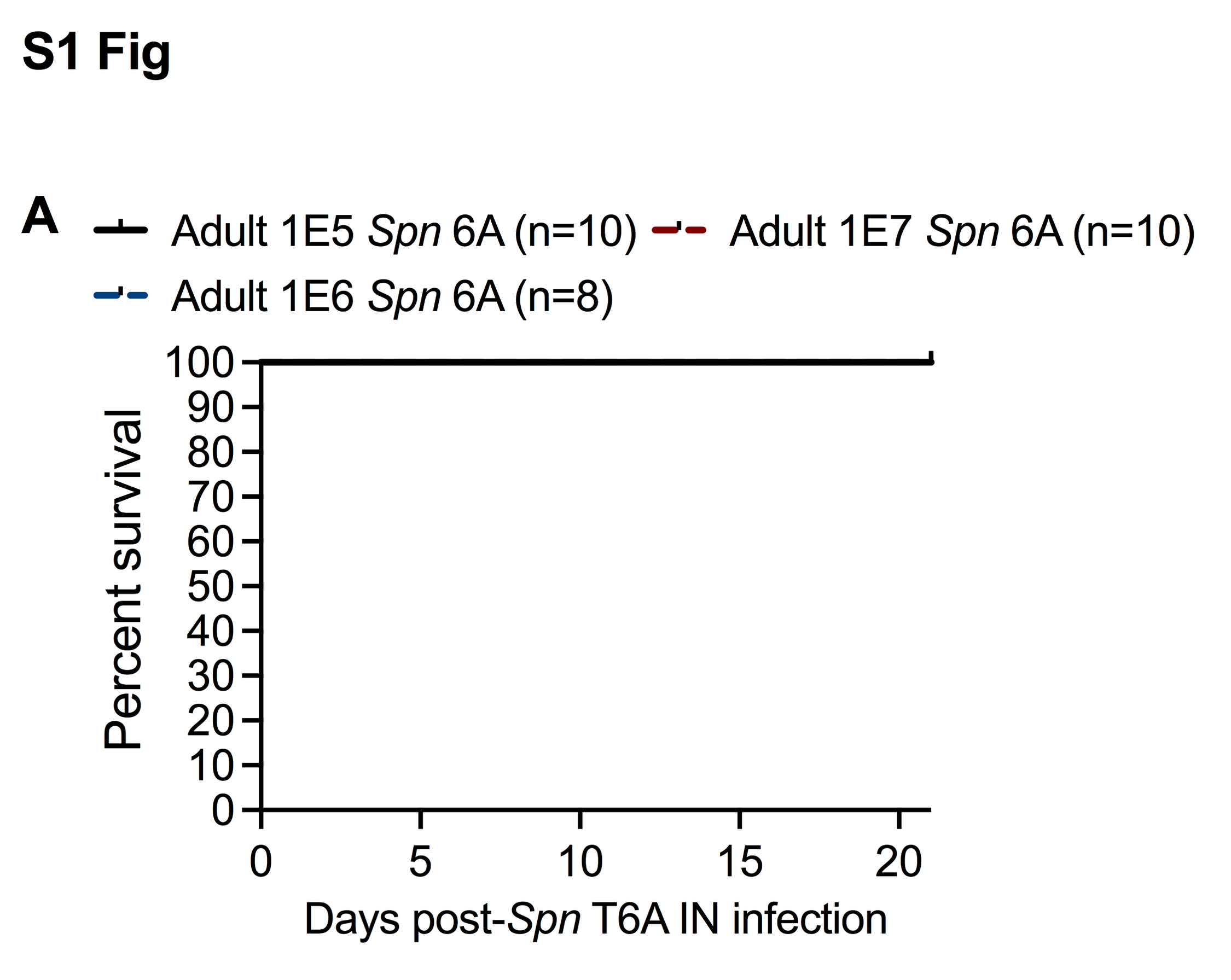

Supplement: S1 Fig — A, Percent survival of adult mice IN infected 1 x 10^5^, 1 x 10^6^ or 1 x 10^7^ CFU of Spn T6A at 21 dpi (n = 8–10). Data are collected from one experiment. (TIF) [file ppat.1012111.s001.tif]

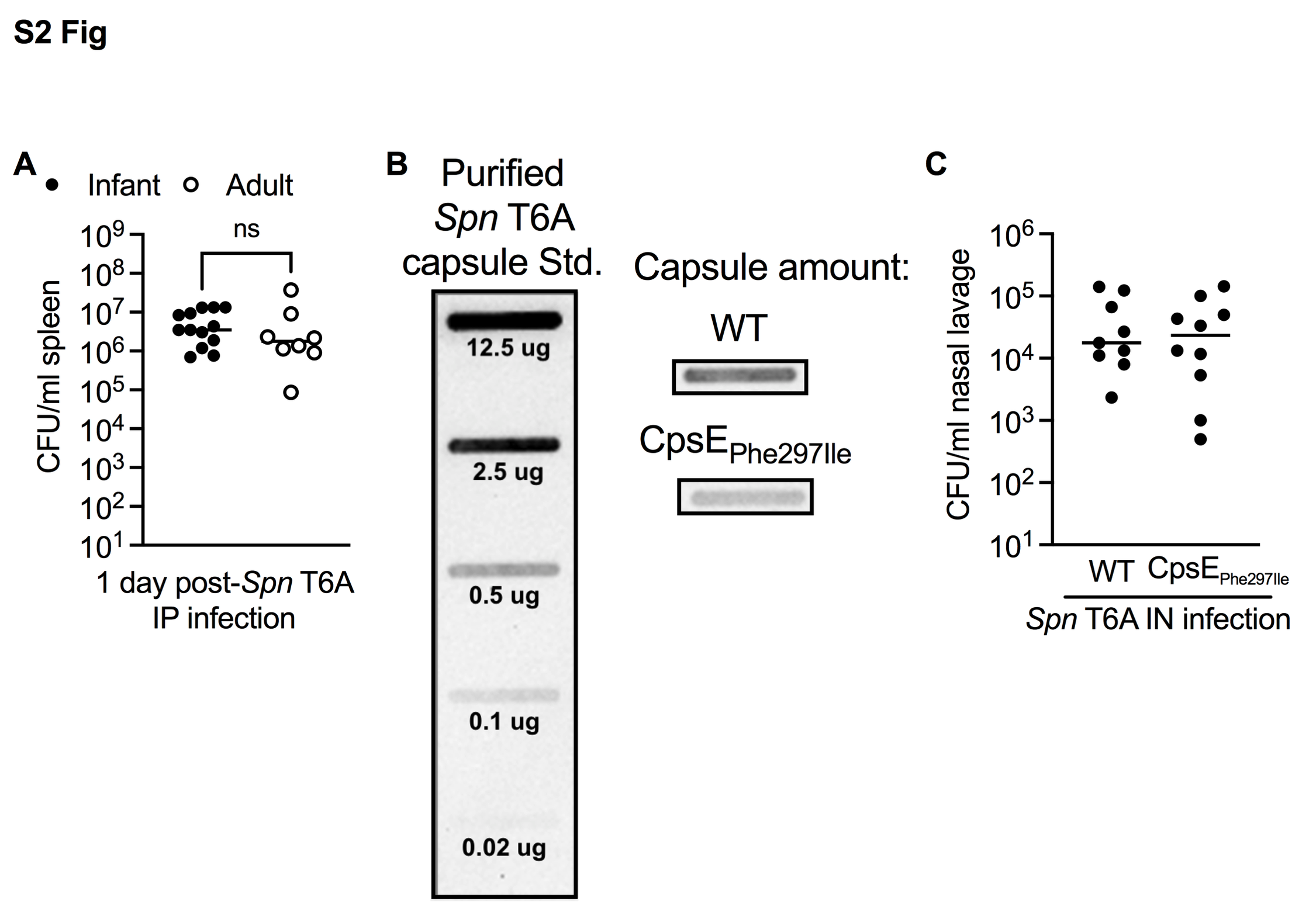

Supplement: S2 Fig — A, Blood CFU from infant and adult mice intraperitoneally (IP) infected with 102 CFU of Spn T6A at 1 dpi (n = 8–13). Statistical significance determined using Mann-Whitney test. ns, not significant. Data represent individual mice with median and are collected from two independent experiments. B, Immunoblot of capsule levels from purified Spn T6A capsule standard (Std.), Spn T6A (WT) and Spn T6A CpsEPhe297Ile mutant. C, Nasal lavage CFU from infant mice IN infected with either Spn T6A (WT) or Spn T6A CpsEPhe297Ile mutant at 14 dpi (n = 9–10). Data represent individual mice with median and are collected from 1–2 experiments. (TIF) [file ppat.1012111.s002.tif]

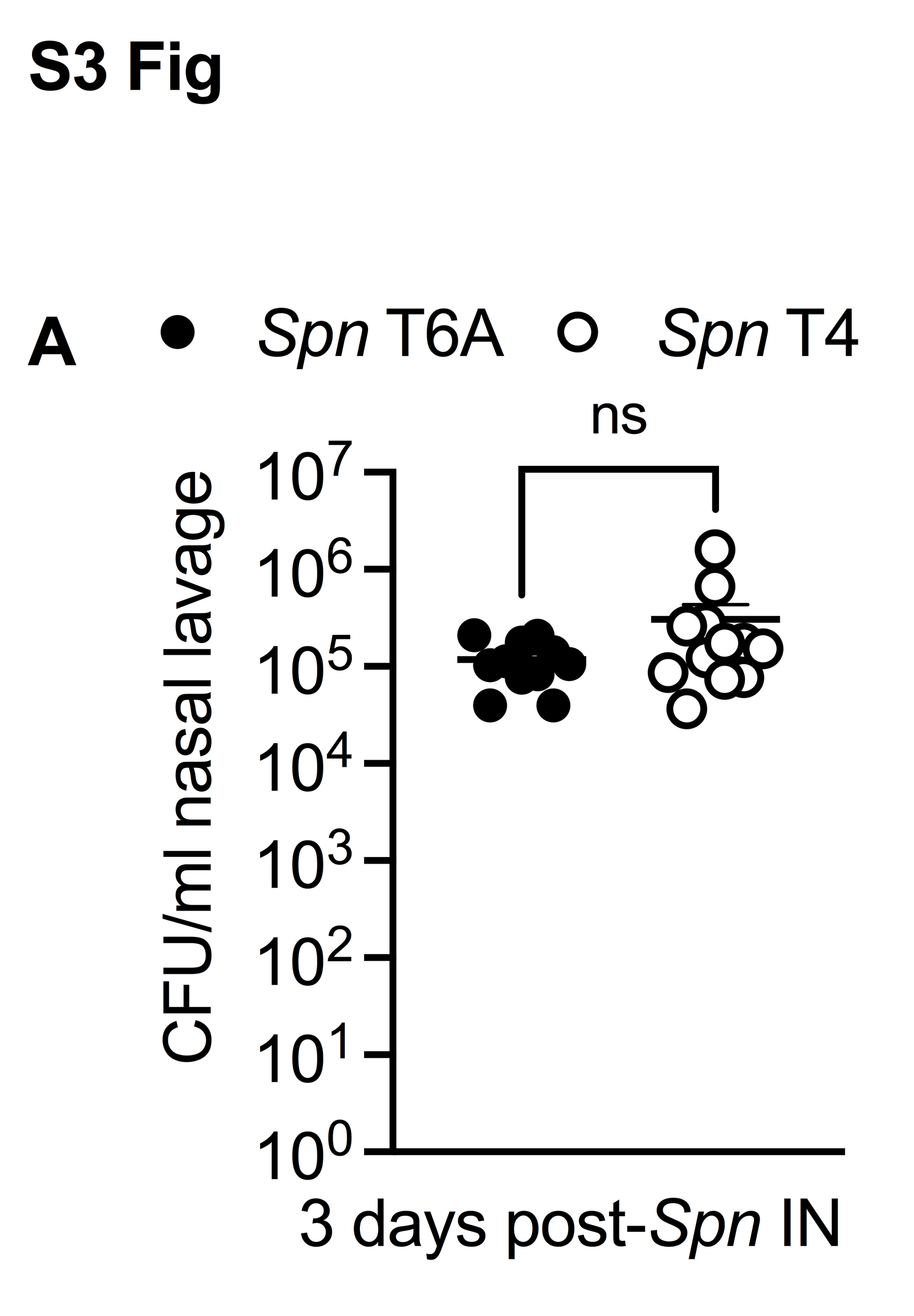

Supplement: S3 Fig — A, Nasal lavage CFU from infant mice IN infected with either Spn T6A or Spn T4 at 3 dpi (n = 11–12). Data represent individual mice with mean ±SEM and are collected from 2 independent experiments. Statistical significance determined using Mann-Whitney test. ns, not significant. (TIF) [file ppat.1012111.s003.tif]

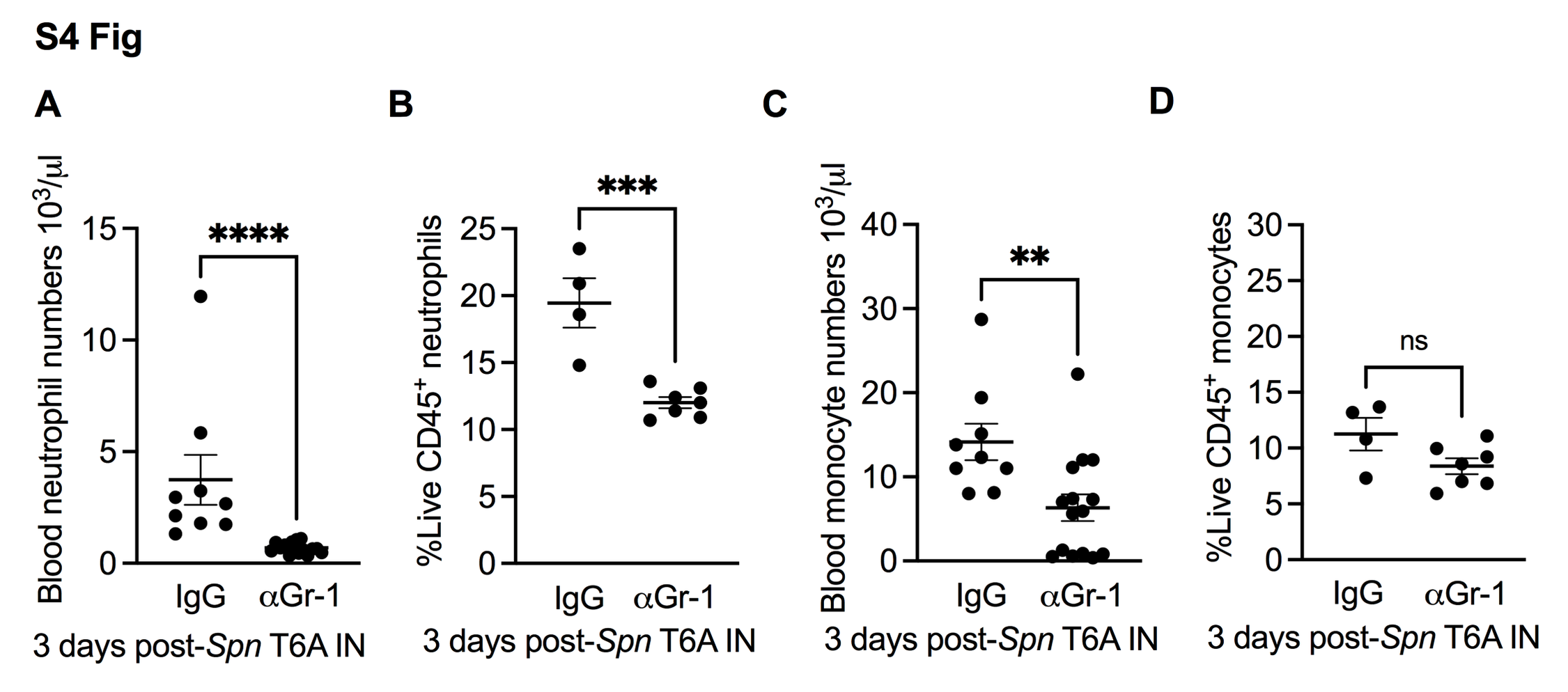

Supplement: S4 Fig — A-D, Infant mice IP treated with IgG isotype control (IgG) or anti-Gr-1 (αGr-1) antibody and IN infected with Spn T6A at 3 dpi (n = 9–12). A, Number of neutrophils in blood. B, Percentage of neutrophils (Live CD45+ CD11b+ Ly6G+) in nasal tissue. Mouse nasal tissue was pooled n = 2–3 per sample. C, Percentage of monocytes in blood. D, Percentage of monocytes (Live CD45+ CD11b+ Ly6G- Ly6C+) in nasal tissue. Mouse nasal tissue was pooled n = 2–3. Data represent individual mice with mean ±SEM and are collected from 2–3 independent experiments. Statistical significance determined using unpaired Students t test or Mann-Whitney test. **, p ≤ 0.01; ***, p ≤ 0.001; ****, p ≤ 0.0001; ns, not significant. (TIF) [file ppat.1012111.s004.tif]

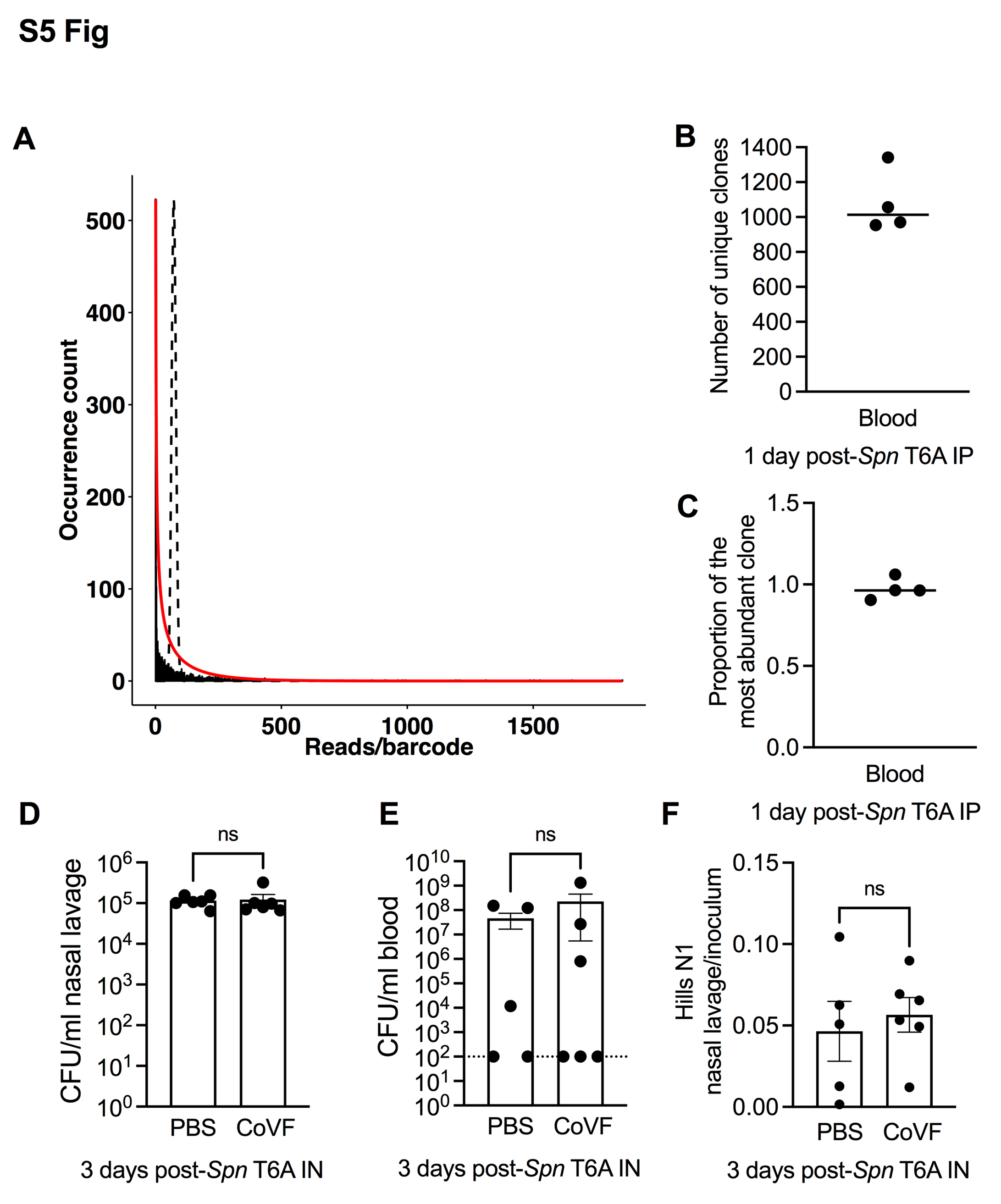

Supplement: S5 Fig — A, Spn T6A molecularly-barcoded library diversity. Histogram indicates the frequency occurrence (Y-axis) of the number of reads per barcode (X-axis). B, Number of unique clones and (C) proportion of most abundant clone in the blood from infant mice IP infected with Spn T6A barcoded library at 1 dpi. Data represent individual mice with median and are collected from one experiment. D, Nasal lavage and (E) blood CFU from infant mice treated IP with dPBS or cobra venom factor (CoVF) and IN infected with Spn T6A barcoded library at 3 dpi (n = 5–6). Data represent individual mice with mean ±SEM and are representative of one experiment. Statistical significance determined using Mann-Whitney test. ns, not significant. F, Hill’s N1 diversity coefficient in nasal lavage samples collected from septic infant mice that were treated IP with dPBS or cobra venom factor (CoVF) and IN infected with Spn T6A barcoded library. Data represent individual mice with mean ±SEM and are collected from two independent experiments. Statistical significance determined using Students t test. ns, not significant. (TIF) [file ppat.1012111.s005.tif]
