# Supplementary material for: Impaired upper respiratory tract barrier function during postnatal development predisposes to invasive pneumococcal disease
Source: PLoS Pathog. 2024 May 8;20(5):e1012111. doi: 10.1371/journal.ppat.1012111 (PMC11078396; doi:10.1371/journal.ppat.1012111)
Supplement: Attachment — Submitted filename: ResponsePlosPathogens.docx [file ppat.1012111.s008.docx]

Reviewers find the manuscript to be on an important topic and the overall premise of impaired barrier function to be potentially valid explanation for enhanced susceptibility of young animals/infants. However, all reviewers felt that the rigor of the work could be improved with inclusion of key controls, better explanation of methodology/presentation of the data, and some additional data.

Author’s response: We want to thank the reviewers for their careful review of our manuscript. Their comments were instrumental in producing a revised and clearer manuscript. We added new data in Figures 3a, 3d, 6d and Supplemental Figures 2b and 3a to support our conclusions. The introduction has been updated to provide context, the discussion has been shortened and the methods section has been modified to provide a more detailed description of the experiments performed.

Reviewer's Responses to Questions

**Part I - Summary**
Please use this section to discuss strengths/weaknesses of study, novelty/significance, general execution and scholarship.

Reviewer #1: Overall, this manuscript provides convincing evidence that barrier development in the URT is a key factor in susceptibility to pneumococcal infection. There is extensive experimental evidence, including several negative results (which are important controls for this study and should remain included in the paper), that support this hypothesis. I was particularly impressed by the human validation cohort, which recapitulated much of the observations made the murine system. The conclusions were well supported by the experimental data and the manuscript was presented in a clear and logical fashion. The methods were well detailed and easy to follow.

Reviewer #2: In this manuscript, Lokken-Toyli et al. investigate the age-dependent mechanisms by which S. pneumoniae crosses the upper respiratory tract (URT) barrier in infants but not in adults. Using a mouse model, they show that age-dependence correlates with an infection-independent increase in URT barrier permeability and delayed clearance of mucosa-adherent bacteria in young mice. This correlates with upregulated expression of genes encoding tight junction proteins in both adult mice and humans.

Reviewer #3: Lokken-Toyli et al. observe an increased mortality and blood CFU to i.n. S. pneumoniae (T6A) infection in infant (d10) vs adult mice and aim at identifying the underlying reasons for age-dependent infection susceptibility. Notably, this observation is reminiscent to the situation in humans, where infants carry S. p. in their throat and develop occult bloodstream infection prior to invasive infection (meningitis, sepsis) whereas adults develop LRT infection pneumoniae followed by bloodstream infection.

The group has previously published a number of articles on the neonatal S. pn. infection model among others to study host transmission (IAI, 2017) and shown that pneumolysin-induced inflammation drives transmission (CHM, 2017), that susceptibility of infant mice is due to a lack of pneumolysin-induced IL-1a secretion (PLoS Pathogens, 2018), that vaccine induced immunity prevents shedding and transmission (mBio, 2017) and that host transmission is determined by the capsule type (mBio, 2018). In particular, they have shown that pathogen-induced downregulation of tight junction proteins facilitates epithelial translocation (Clarke et al., 2011).

Here, they demonstrate (i) that i.n. and less so i.p. infection with Streptococcus pneumoniae T6A is associated with higher mortality and increased spleen and blood CFUs in infant (d10) mice as compared to adult (8-10w) mice, (ii) that bacterial capsule expression is critical for blood CFU and survival after parenteral infection (i.p.) but not nasal colonization in infant mice, (iii) that complement depletion does not affect survival after i.n. infection of adult or infant mice, (iv) that S.p. T4 exhibits an attenuated phenotype after i.p. infection (survival and blood CFU) and that complement provides protection from systemic T4 i.p. infection (blood CFU, survival), (v) that T6A S. pn. Infection i.n. leads to increased chemokine expression and PMN recruitment (but PMN depletion has no effect on colonization nor blood or spleen CFU or survival), (vi) that deletion of the toxin pneumolysin in T6A S. pn. does not lead to differences in colonization, blood or organs CFU or survival after i.n. infection (with a minor protection by Tlr2 and maybe Il-1R1), (vii) that epithelial translocation represents a major bottleneck independent of complement, (viii) that URT tight junction expression increases with age in mice and man (and FITC dextran translocation decreases in mice), (ix) that tight and adherens junctions expression is reduced in neonates but not adults after S.p. 23F infection but neither 4kDa nor 2000kDa FITC dextran translocation is not increased in neonates infected with T6A, (x) that FITC dextran translocation is enhanced postnatally (until day 14) and infection with T6A at 15-16 days after birth does not lead to mortality (at day 10 it is 39%), FITC dextran clearance from the URT mucosa is delayed up to day 14 after birth, (xi) that i.n. T6A infection leads to relatively lower tissue number as compared to mucosal bacteria in adults as compared to infants and (xii) that T6A leads to close epithelial attachment and transepithelial translocation in infant but not adult mice. Thereby, they show that the enhanced susceptibility in infants following i.n. inoculation has something to do with the early course of the infection and is NOT dependent on bacterial capsule or pneumolysin expression, host complement or PMN recruitment (although enhanced). ORT epithelium translocation generally represents a major population bottleneck and epithelial tight junction expression and luminal clearance is lower in infants than adults and further reduced upon infection. Epithelial attachment and translocation of S. pn. are enhanced in infants.

The research topic is highly relevant. In principle, the data are interesting and novel. In the current text version, the story is hard to follow. Many experiments mainly show negative results for host and bacterial factors. Although consistent with the histological images shown, no function data are provided (and are most likely difficult to generate) that would proof the role of reduced tight junction formation for the enhanced barrier permissiveness for S.p. Other difference might contribute as well.

**Part II – Major Issues: Key Experiments Required for Acceptance**
Please use this section to detail the key new experiments or modifications of existing experiments that should be absolutely required to validate study conclusions.

Generally, there should be no more than 3 such required experiments or major modifications for a "Major Revision" recommendation. If more than 3 experiments are necessary to validate the study conclusions, then you are encouraged to recommend "Reject".

Reviewer #1: 1. In the section describing the independence of Spn translocation across the URT barrier in infant mice being independent of capsule type- rather that comparing two different strain backgrounds with different capsule types, the more rigorous experiment would be to utilize capsule switching variants that only differ in their capsule locus.

Author’s response: The goal of this section was to demonstrate that age-dependent differences in barrier function facilitate translocation of different *Spn* strains. For clarity we removed the reference to serotype as the critical factor for invasive *Spn* infection. Capsule has already been established as a critical factor for invasive infection, so this was not a goal of our study.

Reviewer #2: While the findings may be important for understanding S. pneumoniae infection in young children, the methods are not detailed enough to be confident of the relevance of the authors' conclusions. In addition, this study reports on correlations or absence of thereof, and the authors conclusions therefore remain speculative. The discussion would benefit from being more concise.

Author’s response: We have modified the methods and shortened the discussion.

Major points:

Introduction: There is a lack of context to explain what is known, what remains to be investigated and how this study differs from the previous one.

Author’s response: Additional background information has been added to the introduction; lines 108-120.

Figure 1: The IN model needs to be better explained: why 103 CFU in infant mice vs. 105 CFU in adults?

Author’s response: Inoculum dose was calculated for 10x the 50% colonizing dose for infant and adult mice. This has been added to the methods; lines 633-634.

Figure 1b, 1c: How are the CFUs for the dead animals estimated? Based on the most infected live animal? If so, they need to be removed from the statistical analysis (estimation and no proof that it's the real data).

Author’s response: A blood sample could be not collected from animals that were found dead. *Spn* sepsis was confirmed in these animals using splenic cultures, and blood CFUs from these animals are represented in the figure as a red cross and are not included in the statistical analysis.

Figure 1d: Colonization is not similar on day 1, which could partially explain the increase in bacterial counts in the blood of young mice.

Author’s response: Infant mice do not become septic until approximately 3 days post-Spn T6A colonization, a time-point when nasal colonization levels are similar between infant and adult mice. Adult mice remain resistant to developing sepsis despite reaching similar CFUs at 3 days post-*Spn* infection.

Supplementary Figure 2b: This WB is inconclusive. A reference Spn protein is needed to normalize the amount of capsule. Please show both lines on the same blot to compare the size, or at least with a common marker size.

Author’s response: To account for sample loading, total protein concentration was normalized for each strain. A purified type 6A capsular polysaccharide standard was loaded alongside samples, all on the same blot, in order to calculate capsule amounts. The standard image is now included in Supplementary Figure 2b. The methods have also been updated; lines 654-678.

Figure 2D: A control is missing to know if CovF works: IP infection?

Author’s response: We previously demonstrated the efficacy of CovF treatment using a T6A strain that expresses 60% less capsule (PMID: 37540710). Additionally, in our study, we validated the efficacy of CovF treatment using Spn T4 strain (Figure 3c).

Figure 2E: Were T6A and T4 experiments done in the same experiment? What are the CFUs in nasal lavage for T4?

Author’s response: The survival experiments for T6A and T4 were not done in the same experiment. Nasal lavages for 3 days post-infection are now provided in Supplemental Figure 3a.

Figure 2H: Why is the x-axis different from 2e (8 vs 14 days)? Did you follow the animals up to 14 days pi? Would you expect mice with PBS to die at 5 days post infection (compared to no death in Figure 2e)?

Author’s response: We used the time-point of 8 days post-Spn IN infection for all CoVF experiments considering the majority of infant mice become septic 3-5 days post-Spn (Figure 1A). It is possible that the stress of being handled and IP inoculated with PBS could contribute to the death observed at 5 days post infection, although this is at a significantly reduced rate compared to CoVF treated infants.

Page 10, lines 202 to 205. It can't be concluded that disruption of the URT barrier function allows translocation of Spn independent of serotype. An experiment comparing infant and adult infection with Spn T4 is missing. Complement-independent immune mechanisms may also be involved.

Author’s response: As suggested, survival curves using adult mice colonized with Spn T4 (new Figure 3a) and Spn T4 colonized adults treated with either PBS or CoVF (new Figure 3d) has been added. Together, this data demonstrates that in infant mice, but not adults, Spn T4 can translocate across the URT barrier; however due to increased sensitivity to complement-mediated phagocytosis Spn T4 is rapidly cleared from the bloodstream of infant mice.

Fig 4e: Tlr2-/- mice are more susceptible to infection than wt. What is the author’s interpretation?

Author’s response: Considering the time to sepsis for WT and TLR2-deficient infant mice is similar, we concluded that TLR2 does not impact the initial Spn translocation from the URT of infant mice.

pp13/14 and p28 : Even if already published, the barcode library is not sufficiently described here. How many individual barcodes are there in the library?

Author’s response: There are 3060 clones/individual barcodes in the Spn T6A library. The methods have been updated to include construction of the barcode library, lines 577-587.

Please show the inoculum to compare what is present and what is missing after infection. Fig 5a: What is the proportion of clones conserved in nasal lavage compared to the inoculum?

Author’s response: After taking the number of sequencing reads and rarefaction into account, the inoculum consisted of 1728 clones. The average proportion of clones detected in the nasal lavage at 3 dpi versus the inoculum is 20%.

Fig 5c: one information is missing: is the most abundant clone the same in nasal lavage and blood or are there different clones?

Author’s response: The most abundant clone in the nasal lavage does not necessarily represent the identity of the most abundant clone in the blood. Additional graphs showing the proportion of the most abundant clone in the nasal lavage and its corresponding proportion in the blood have been added (now Figure 6d).

Fig S4b: What is the inoculum? If there are 103 CFU, how is it possible to have more than 1000 unique clones? How is Hill's N1 calculated and what does it mean? Again, a more detailed description of the method will help.

Author’s response: The inoculum for the Spn T6A library was 10^5 CFU for IN infection and 10^4 for IP infection and is described in the methods; lines 636-638.

The Hill’s N1, otherwise known as the clonal diversity index (H), was calculated as H=−∑pi.ln(pi) where *p*_i_ denotes the proportion of the population made up of the clone *i*. This calculation was added to the methods; lines 614-616.

Fig 6&7: These are nice correlations between the expression of genes encoding tight junction proteins and susceptibility to infection. The fact that fluorescent dyes cross the barrier more efficiently in young mice than in adults (Fig 6d) supports this correlation. Ideally, one would expect to confirm experimentally that this is the main mechanism (compensation of barrier permeability in young, decrease of barrier effect in adults…). However, the authors are cautious in their interpretation ("suggested", "indicated"...).

Author’s response: As the reviewer points out, experimental modulation of barrier function is empirically challenging.

Fig. 7a: The number of points is very different at different ages. One would expect different results if n were the same for all conditions.

Author’s response: The finite sample sizes are taken into account in the Kruskal-Wallis post hoc Dunn’s multiple comparisons test and resulting P value that shows significance.

Reviewer #3:

1. The data presented are complex and the logic of their presentation is hard to follow. Restructure the presentation of the findings, maybe move some panels to the supplement, provide clear conclusions from each figure,

Author’s response: The data in Figure 2 has been divided into two figures (Figures 2 and 3) and the conclusions for both Figures have been clarified for easier interpretation.

2. Comment on the reason for the use of the different S. pn. strains (T4,. T6A, 23F) (or repeat experiments with the similar S.p. strain to allow a comparative analysis) to make it easier to follow the logic of the presented data also for non-S. pn. specialists.

Author’s response: Spn strains are variable in their ability to cause invasive infection. Different Spn strains were used to determine the age-dependent host factors that drive increased susceptibility to invasive Spn infection in infant mice

3. Always show in the figure whether infection are i.n. or parenteral (i.p.).

Author’s response: Done as suggested.

4. Quantify the histological data in figure 7e and f.

Author’s response: Quantification of Spn in the histological sections, now Figure 8e-f, is challenging considering there was limited staining of Spn in adult mice. We quantified bacterial localization by assessing CFUs from the lumen (i.e. nasal lavage) versus tissue from infant and adult mice (now Figure 8d).

5. Please reconcile the findiongs in Fig. 6h and I with the recent publication Clarke et al., 2011.

Author’s response: In the Clarke et al. publication, the authors demonstrate that Spn colonization of adult mice leads to a downregulation of claudins compared to PBS control adult mice. These results do not take into account the impact of age. The data presented in our study, now Figure 7h and I, compare the transcriptional response of Spn-infected infants to Spn-infected adult mice.

6. Page 7, “higher blood and spleen CFU despite similar levels of colonization of URT”. The data show a significant difference at day 2 p.i..

Author’s response: As noted above, infant mice do not become septic until approximately 3 days post-Spn T6A colonization, a time-point when nasal colonization levels are similar between infant and adult mice. Despite reaching similar levels as infant mice, adult mice remain resistant to developing Spn sepsis.

7. Please discuss why infant mice (and humans children) might express lower tight junction levels. What could be the benefit of expressing lower levels than in adults.

Author’s response: In the discussion, we speculate that compromised barrier function mediated by reduced expression of tight junction genes might facilitate the physical maturation of the expanding airway, lines 509-513. Additionally, increased barrier permeability in infants might allow for sampling of URT contents to promote immune maturation and tolerance, lines 526-533.

**Part III – Minor Issues: Editorial and Data Presentation Modifications**
Please use this section for editorial suggestions as well as relatively minor modifications of existing data that would enhance clarity.

Reviewer #1: Overall, I thought the paper was well-written and studies were well designed. This paper provides substantial value to the field. There were a few parts throughout that needed some clarification, and maybe some points to consider in the discussion.

Line 112: should read, “Streptococcus”

Author’s response: The text has been modified, now line 122.

Line 129: I don’t really feel like this is adding anything considering that 10^5 CFU is still considered sub-lethal for adult mice. Is 10^3 CFU still considered sub-lethal for pups?

Author’s response: Inoculum dose was calculated for 10x the 50% colonizing dose for infant and adult mice.

Line 149: Spn capsule also enhances intracellular survival and vascular endothelial cell translocation, important reference to add (Brissac et al., 2021).

Author’s response: Reference has been added; lines 157-159.

Line 206: This statement conflicts with the section header. The section header states that the phenotype is independent of serotype, but the last sentence directly states it is in fact dependent on serotype.

Author’s response: For clarity, we revised the conclusions statement (lines 211-218) and changed the section header.


Line 227: Doesn’t Gr1 also target monocytes? Is there a reason why an antibody targeting specifically neutrophils (i.e., Ly6G) wasn’t used?

Author’s response: The anti-Gr-1 antibody was more thorough in mucosal neutrophil depletion than the neutrophil specific anti-Ly6G antibody, despite being less specific considering monocytes also express Gr-1. We accounted for a possible effect of monocytes in Figure 4i by using CCR2-deficient mice.

Line 241: Phagocytic ROS production has been shown to be essential for bacterial restriction in some infection models (i.e., pulmonary) but not others (skin) using group A Streptococcus (Lei et al., 2017), so it is possible that a similar mechanism may be at play and may be part of why you don’t see a phenotype in the URT.

Author’s response: Considering ROS has been shown to be important during pulmonary Spn infection (PMID: 18202350), we felt we needed to determine its role in URT infection.

Line 310- It would be beneficial to state in either the results or the discussion whether altered regulation of any of the key pneumococcal receptors were identified in the RNA-seq datasets.

Author’s response: As mentioned in the discussion (lines 525-526), the age-dependent regulation of potential pneumococcal receptors is an area of future investigation and beyond the scope of this study.

Figure 5c: May benefit from color-coding each pair of points for visibility.

Author’s response: Done as suggested.

In Figure 6- I was surprised there was no difference in the permeability following pneumococcal challenge (Fig 6J). Is this perhaps strain specific? This is in contrast to previous reports whereby colonization of the epithelium by pneumococcus downregulates tight junction components (PMID: 21575911). The most likely explanation being that the tight junctions in the infant animals is already at such a low level, the pneumococcal colonization does not drive it down further?

Author’s response: Our previous study (PMID: 21575911) showed that Spn colonization led to the downregulation of two tight junction proteins, claudin 7 and 10, in Spn colonized adult mice compared to mock infected adult controls; therefore the impact of age was not accounted for. In this study, we found that claudin 10 expression was significantly reduced in both mock- and Spn-infected infant mice compared to adult controls (now Figure 7a and h). As the reviewer stated, it is likely that the age-dependent regulation of tight junction expression outweighs any additional influence of Spn colonization on barrier permeability in infant mice.

Reviewer #2:

Write IP or IN directly on each graph to make it easier to analyze the figures.

Author’s response: Done as suggested.

Supp Fig 2a: "Spleen" is written on the graph, but "Blood" is written in the legend.

Author’s response: The legend has been updated to read “Spleen”.

Fig 3 Anti-GR-1 RB6-8C5 is known to deplete cells other than neutrophils (monocytes, dendritic cells... see Daley JM et al, J Leukoc Biol. 2008). Here you show the lack of role of neutrophils and other cells. If you want to focus on neutrophils only, you will need to choose a different antibody or modify your text to reflect the antibody's property.

Author’s response: As noted above, the anti-Gr-1 antibody was more thorough in mucosal neutrophil depletion than the anti-Ly6G, but was less specific considering monocytes also express Gr-1. We accounted for a possible effect of monocytes in Figure 4i by using CCR2-deficient mice.

Reviewer #3: Minor points:
1. When FITC dextran is given intranasally and plasma FITC is used as read out, how can translocation through the gastrointestinal tract be excluded as a reason for differences?

Author’s response: To minimize the potential contribution of FITC-dextran translocation from the gastrointestinal tract, the FITC-dextran was titrated and administered using both an oral and IN route to determine the optimal IN dose.

2. Also provide absolute read counts for figure 3a-c and absolute cell counts for Figure 3d and e (see text line 221 “numbers of”).

Author’s response: We have replaced “numbers of” to “proportion of”, new line 233.

3. Last sentence page 17 1st paragraph “…that berrier function is a consequence of postnatal development and independent of colonization” and the first sentence in the discussion that “age-dependent disruption … following colonization of infant mice”. Please reconcile with the findings shown in Fig. 6h and i.

Author’s response: For clarity the first sentence of the discussion was modified, lines 416-417.
